# On the efficiency of HIV transmission: Insights through discrete time HIV models

**DOI:** 10.1371/journal.pone.0222574

**Published:** 2019-09-18

**Authors:** Sarudzai P. Showa, Farai Nyabadza, Senelani D. Hove-Musekwa

**Affiliations:** 1 Department of Applied Mathematics, National University of Science and Technology, Bulawayo, Zimbabwe; 2 Department of Mathematics and Applied Mathematics, Auckland Park Campus, University of Johannesburg, Johannesburg, South Africa; Cincinnati Children’s Hospital Medical Center, UNITED STATES

## Abstract

There are different views on which of the two forms of viral spread is more efficient *in vivo* between cell-free and cell-associated virus. In this study, discrete time human immunodeficiency virus models are formulated and analysed with the goal of determining the form of viral spread that is more efficient *in vivo*. It is shown that on its own, cell-free viral spread cannot sustain an infection owing to the low infectivity of cell-free virus and cell-associated virus can sustain an infection because of the high infectivity of cell-associated virus. When acting concurrently, cell-associated virus is more efficient in spreading the infection upon exposure to the virus. However, in the long term, the two forms of viral spread contribute almost equally. Both forms of viral spread are shown to be able to initiate an infection.

## Introduction

Human immunodeficiency virus (HIV) is transmitted when infected blood, semen, vaginal fluids, or breast milk enter another person’s body. Once the virus enters the body, it can spread through target cells as a free viral particle or in cell-associated form [1, 2]. Cell-free virus, is the plasma virus, whereas cell-associated virus, is the intracellular progeny virion that has been produced but not yet budded off the manufacturing T-cell [3]. In cell-associated viral spread, the processes of budding, attachment and entry, proceed quickly at the sites of cell-to-cell contact. This partially protects the virus from the hostile extracellular environment and also concentrates the viral particles at the sites of infection [3]. In cell-free viral spread, the replicated viruses bud off from the producer cell, the virus has to diffuse and find a CD4 receptor on the CD4^+^ T cell, attach to the cell and finally enter the cell. Infected blood, semen, vaginal secretions, and breast milk contain both cell-free and cell-associated virus [3–5].

*In vitro* models have shown that cell-associated viral spread is more efficient than cell-free viral spread [2, 6–8]. However, there are different views on which of these forms of viral spread is efficient *in vivo* [9–12]. Understanding the mechanisms employed by the virus to spread within a host is of vital importance as this would provide useful information on treatment and vaccine development [13].

The majority of HIV transmissions occur through male-to-female transmission and the source of viral spread on this transmission route is not known (cell-free or cell-associated virus) [3, 4]. It has been shown that the levels of cell-free virus in blood and semen correlates with infectiousness [14, 15], a result that suggests that cell-free virus contributes significantly to transmission. However, transmission can also occur from individuals with undetectable cell-free virus [16], suggesting that cell-associated virus may have initiated the transmission.

Another common route of transmission is mother-to-child transmission. On this route of transmission, it was shown that the risk of HIV transmission was highly correlated with cell-associated viral load [17], a result that suggests that cell-associated virus contributes significantly to the spread of the infection. In male-to-male transmission, it has been observed that infectiousness correlates with cell-associated viral levels. There is a possibility that the form of virus efficient in transmitting the infection may vary among different routes of transmission. However, different views were obtained from sequence and phylogenetic studies [18–20], results that may lead to the conclusion that the form of transmission efficient, may not depend on the route of infection.

Even though there is growing evidence on the role of cell-associated virus in spreading the infection [10], this infection pathway has received little research attention [4] and thus its relative contributions remain elusive. Moreover, most experiments on protection from HIV acquisition are done using cell-free virus [3, 4] and thus there is need to find the contribution of cell-associated virus in spreading the infection and determine whether the outcomes of these experiments would change if cell-associated transmission pathway is also considered.

Mathematical modelling has been used as a basic tool in understanding the interplay between the HIV pathogen and the immune system and remains the mainstay in this field of research. The study [21] was one of the earliest work modelling the within host dynamics of the HIV infection. The work considered the free viral particles, susceptible and infected CD4^+^ T cells. Many pieces of work expanded this model to include the time delay in cell infection and viral spread and immune response, see for example [22–24] and the references therein. Several studies considered the two forms of HIV spread with the goal of determining the infection dynamics associated with the two forms of viral spread [25–28].

However, few studies have attempted to determine the relative contribution of each of the two forms of HIV spread within the host. The first study that aimed at finding the relative contribution of the different modes of viral spread is given in [29]. The study concluded that cell-to-cell viral spread was more efficient than cell-free viral spread. In 2007, another study [2] also came to the same conclusion. In the study [30], a model that considered susceptible and infected cells was formulated and fitted to the data of the study [2]. The study concluded that the two forms of viral spread contribute equally to viral spread thereby producing conflicting results to the previous results. A study that used an experimental-mathematical approach to elucidate the roles of the two forms of viral spread is given in the study [31]. The study concluded that cell-to-cell viral spread contributes over 60% of virus infection.

In this study, discrete time models which incorporate the life cycle of HIV are developed with the goal of determining the form of viral spread most efficient *in vivo*. Transmission of the infection maybe influenced by the presence of other sexually transmitted infections (STIs) that result in the inflammation or ulceration of the genital mucosa, type of sexual contact, genetic background of the recipient, circumcision status and the amount of infectious virus particles present in the inoculum [4, 32]. The models generalize across all transmission routes and do not include genetic background, STI infection status and any form of intervention. We model situations where the virus has been deposited into an uninfected individual either through sexual contact, breast milk or blood transfusion. This work is different from the previous work [30, 31] in that, models that consider the whole infection cycle of the virus are developed.

*In vitro* models have shown that cell-associated viral spread is more efficient than cell-free viral spread, in order to determine the form of virus more efficient in vivo, we formulated and analysed mathematical models that considered the two forms of viral spread. Separate models are developed for each mode of viral spread and the models are analyzed using dynamical systems theory to determine if any form of viral spread can sustain the infection on its own and also identify the infection cycle characteristics that determine the fate of each form of viral spread. A model that considers both forms of viral spread simultaneously is also formulated and analysed to compare these two forms of viral spread *in vivo*.

## Results

### Cell-free viral spread cannot sustain an infection

**Fig 1:**
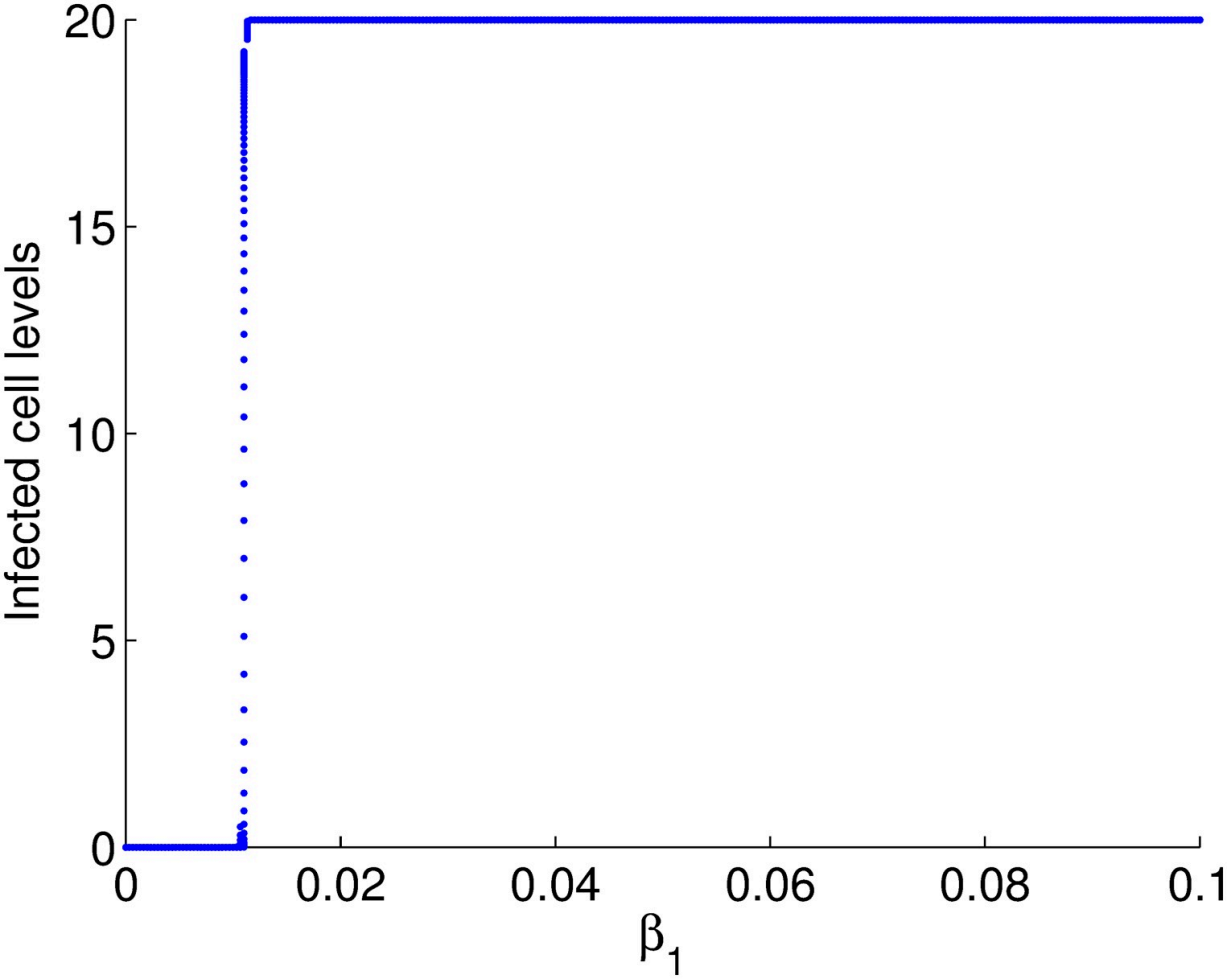
Bifurcation diagram of the cell levels against *β*_1_.

We set out to establish if cell-free viral spread can sustain an infection on its own. We developed a mathematical model that considered cell-free viral spread exclusively, Eqs (1)–(6). Using a bifurcation diagram and parameters obtained from literature on HIV infection, we observed that the disease free equilibrium exist and is unique for the cell-free transmission rate, *β*_1_ less than 0.01. The bifurcation diagram of the cell levels against the transmission parameter is given in Fig 1.

The infectivity of cell-free virus is in the order of 10^−5^ [33] and it can be seen from the bifurcation diagram that if *β*_1_ is within this range, the disease free equilibrium is stable. These results imply that for any biologically feasible parameter set, infection through cell-free virus dies out. We therefore conclude that cell-free viral spread can not sustain an infection on its own because of the low infectivity of cell-free virus.

**Fig 2:**
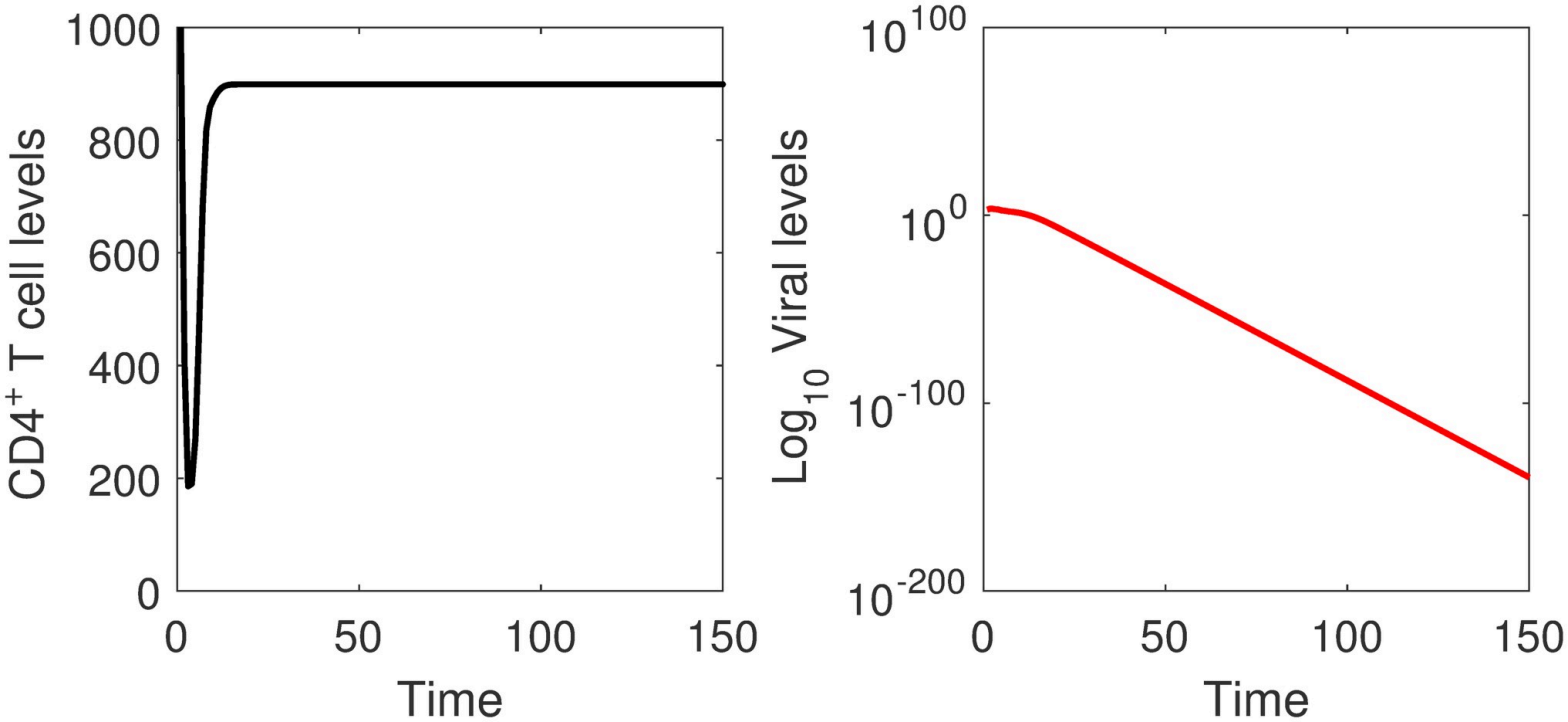
Simulations of the cell-free viral spread model. The viral levels approach zero and the CD4^+^ T cell populations converge to a non-zero steady state as time increases. The infection dies out on its own.

The result that cell-free viral spread does not spread efficiently is consistent with experimental observations in *in vitro* models [6–8]. It has been observed that the low infectivity to a particle ratio of the virus could not explain the efficient spreading in tissue cultures [8, 29, 34]. The virus is also able to spread in tissue cultures despite the presence of neutralizing antibodies that completely block cell-free viral spread [35], meaning that there is some form of transmission besides cell-free viral spread that will be spreading the infection. Numerical simulations for viral and uninfected cell levels are given in Fig 2.

**Fig 3:**
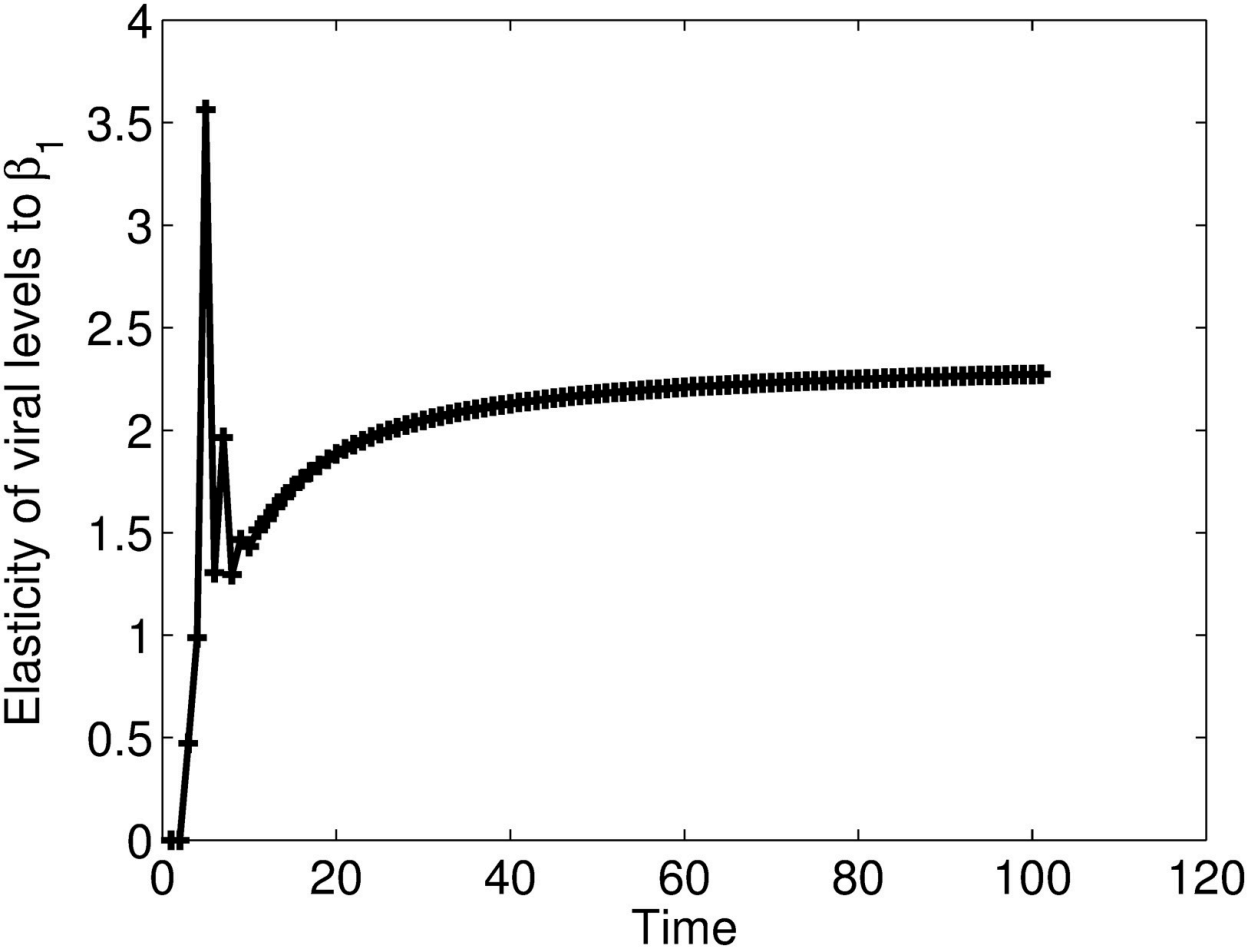
Elasticities of the viral levels to *β*_1_. The results show that the viral level is significantly and positively elastic to *β*_1_. Increases in this parameter will result in increases in viral levels.

Results in Fig 2, suggest that cell-free virus does not spread efficiently since the viral levels are approaching zero with time. The CD4^+^ T cell levels decrease during the first few days of the infection but later stabilize at normal levels. The parameter *ϕ*, the number of virus particles produced per infected cell, seems to be important in determining the outcome of cell-free viral spread. Increasing this parameter to unrealistic values, changes the outcome of cell-free viral spread. A result that shows that the disease equilibrium will only exist for unrealistic parameter values. In Fig 3, we give the elasticity values of the viral levels to *β*_1_. The results show that the viral level is positively elastic to *β*_1_.

**Fig 4:**
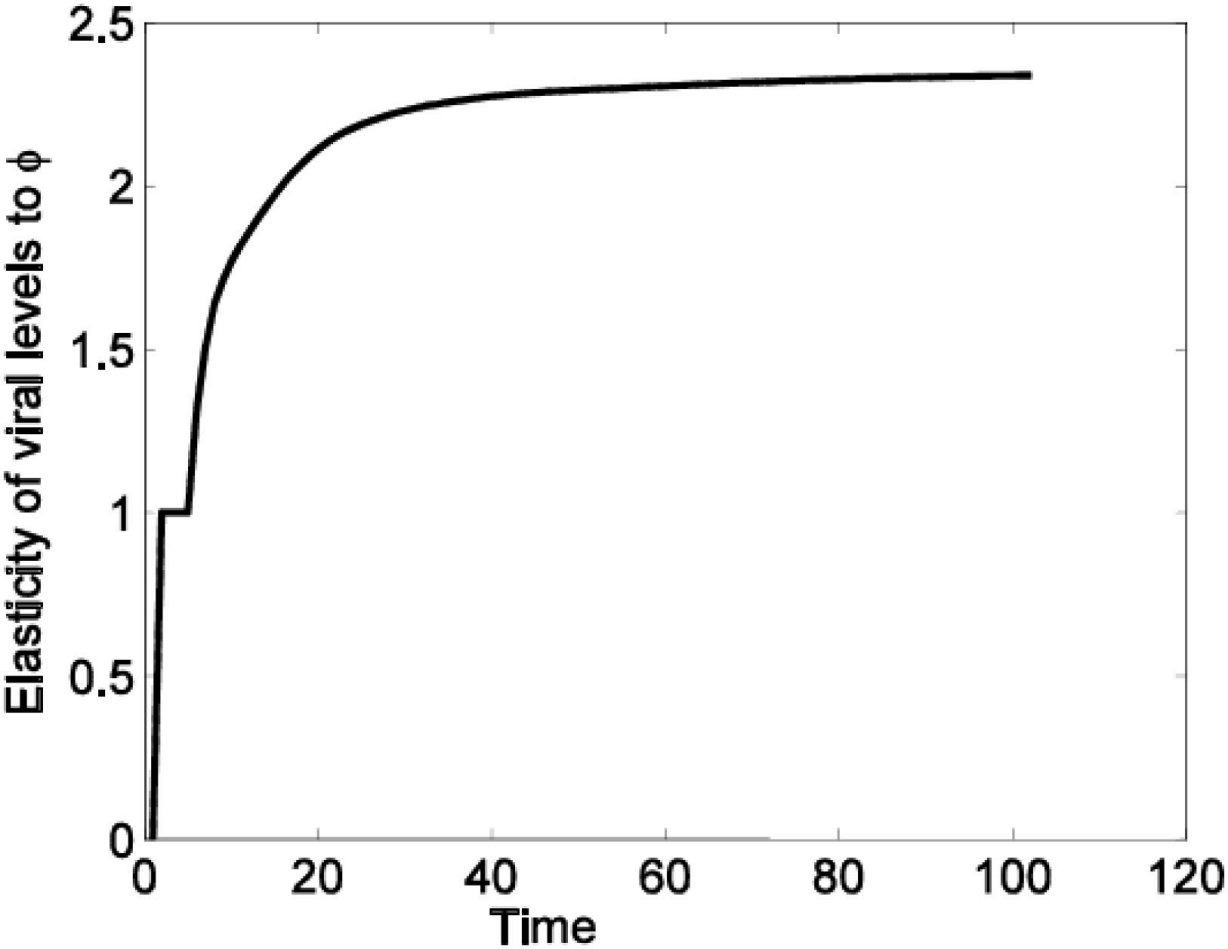
Elasticities of the viral levels to *ϕ*. The results show that the viral level is significantly and positively elastic to *ϕ* and thus increases in this parameter will result in increases in viral levels.

In Fig 4, we give elasticities of viral levels to the parameter *ϕ*.

Results of Figs 3 and 4 show that increases in *β*_1_ and *ϕ* values will result in increases in the viral levels. Results from the above analysis indicate that cell-free viral spread can not sustain the infection on it’s own. However, the fact that cell-free virus could initiate an infection means that this form of viral spread should also be considered in the designing of prevention and intervention strategies. This is so because, even if the cell-free model indicates that the viral levels will approach zero with time, this may not be the case *in vivo* given the many mechanisms such mutations and the latent reservoir, which the virus employ *in vivo* and have not considered in this model.

### Cell-to-cell viral spread cannot sustain an infection for small values of the transmission parameter

**Fig 5:**
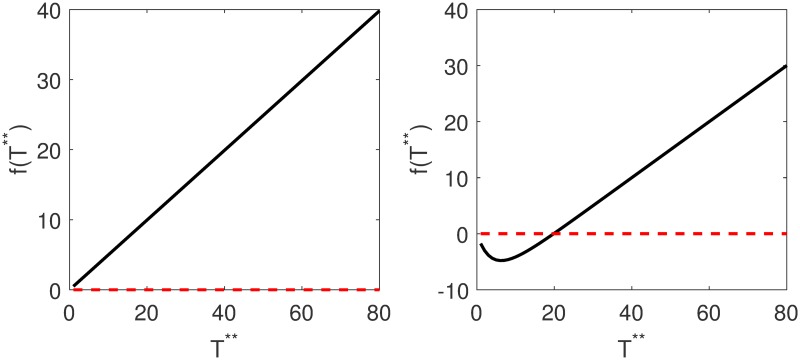
Plots of *f*(T**) against T** where T** is the equilibrium value of infected cells. The parameters used were *β*_2_ = 0.0000024 for Fig 5 a and *β*_2_ = 0.024 for Fig 5 b. The graph does not cross the zero line for small values of *β*_2_ and crosses the zero line for large values of *β*_2_ and thus there is no solution for small values of the transmission parameter. Existence of the disease equilibrium depends on the rate at which cells are infected. *f*(T**) is given in Eq 19.

In order to check if cell-to-cell viral spread could sustain an infection on its own, we developed a mathematical model that considered cell-to-cell viral spread exclusively, Eqs (10)–(15). Using the parameters obtained from literature, we observed that existence of the disease equilibrium depends on *β*_2_, the infectivity (transmission rate) of cell-associated virus as shown in Fig 5.

**Fig 6:**
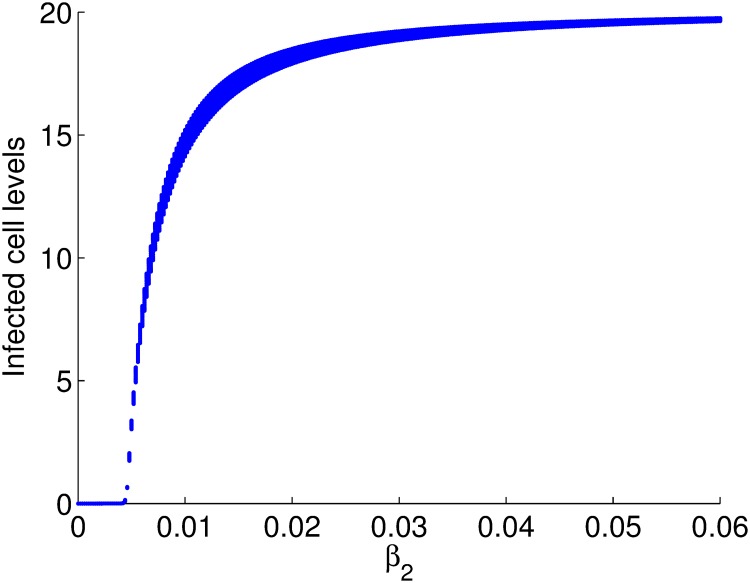
Bifurcation diagram for cell levels against *β*_2_. The disease free equilibrium is stable for *β*_2_ less than 0.005 and unstable for *β*_2_ ≥ 0.005 where the endemic equilibrium become stable. Time is measured in 0.5 days.

We plot the bifurcation diagram of infected cells against the transmission parameter *β*_2_ and obtained the diagram in Fig 6, which confirms the results obtained in Fig 5.

**Fig 7:**
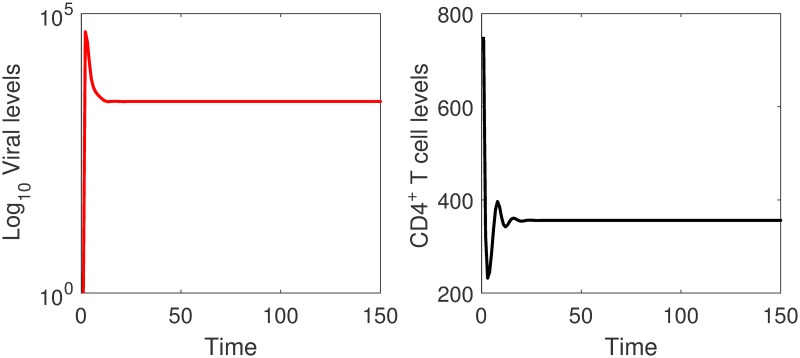
Simulations of the cell-associated viral spread model. The viral and the CD4^+^ T cell levels converge to non-zero steady states. Transmission through cell-associated virus will result in an endermic equilibrium state. *β*_2_ = 0.024.

In Fig 7, simulations of the cell-associated viral spread model are given. It can be seen that cell-associated viral spread can spread the infection efficiently for large values of the transmission parameter.

**Fig 8:**
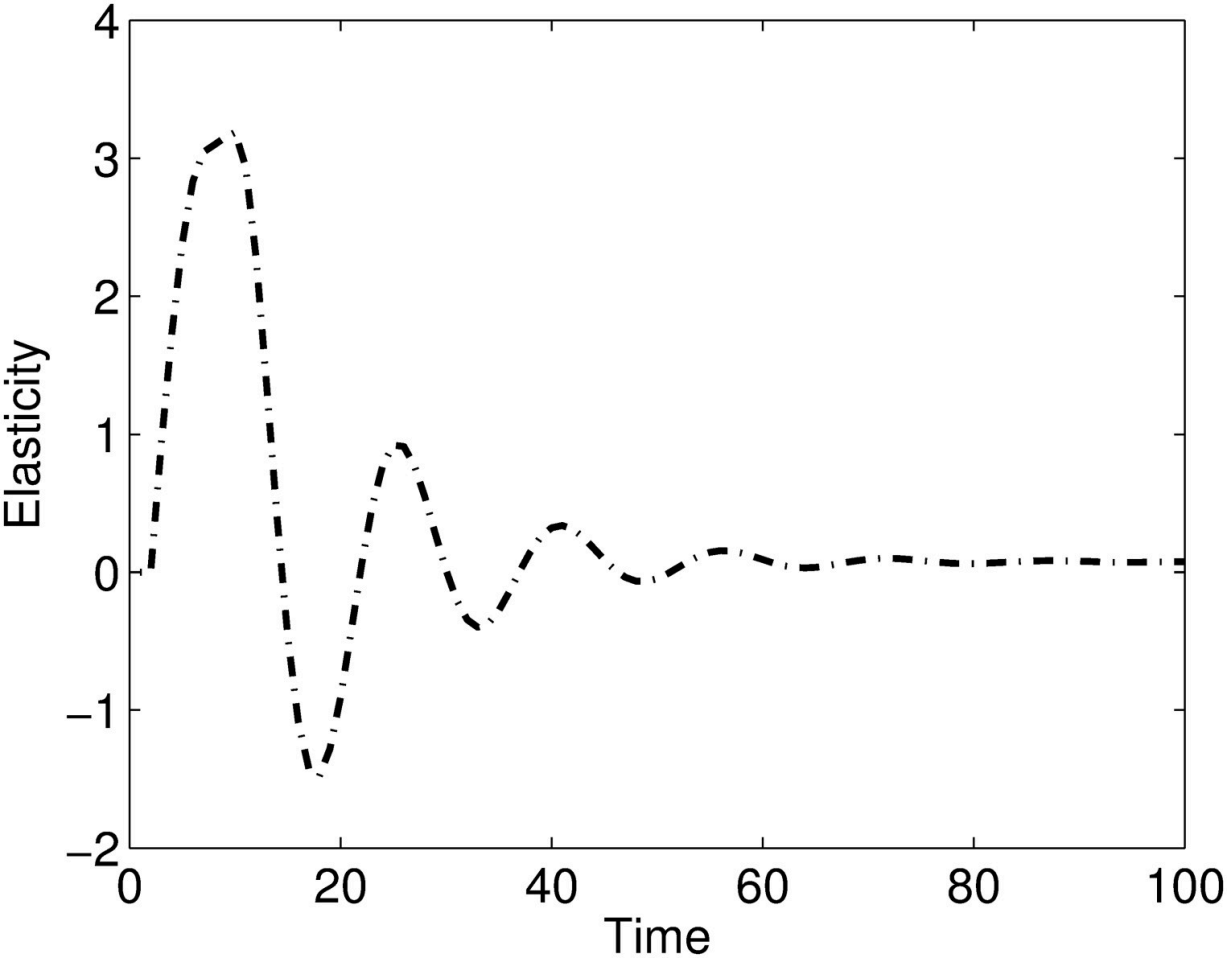
A plot of elasticities of the viral levels to *β*_2_. The viral levels are sensitive to the transmission parameter *β*_2_ during the early days of the infection and as the infection progresses the viral levels become weakly sensitive to the transmission parameter.

The elasticities of the viral levels to the transmission parameter *β*_2_ are given in Fig 8. The results show that the viral level is significantly elastic to *β*_2_ during the early days of the infection and weakly elastic to *β*_2_ as the infection progresses. Thus we can predict that cell-associated transmission parameter *β*_2_ is more important for infection transmission and is not that important in an established infection.

### Cell-to-cell viral spread is more efficient in spreading the virus

**Fig 9:**
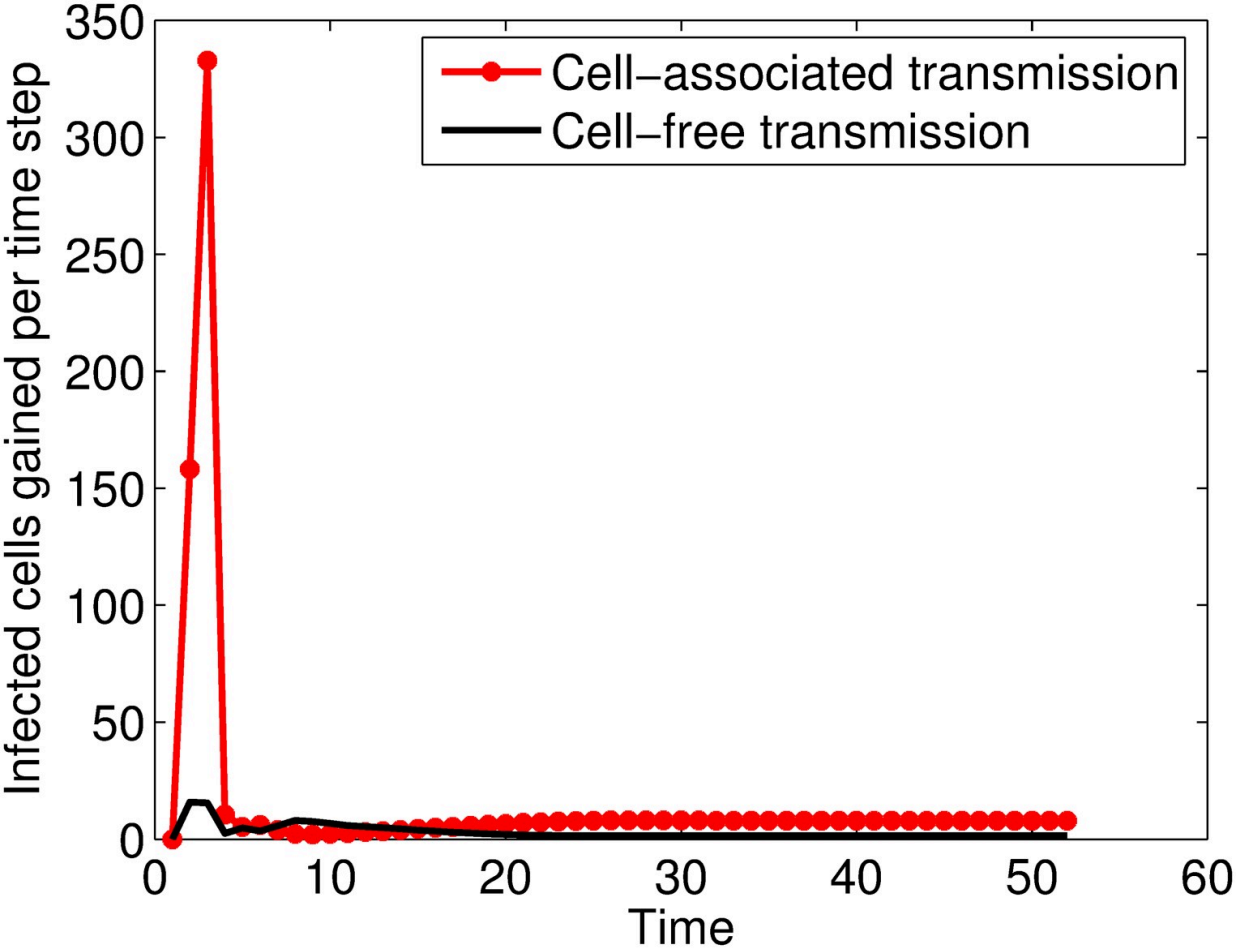
Plots of the infected cells gained per time step against time. Time is measured in 0.5 days.

In an HIV infection, both forms of transmission occur concurrently [1, 2] such that the within-host HIV infection is best modelled by a model that considers both forms of transmission. In this scenario, a cell has to escape infection by cell-free virus and cell-associated virus for it to remain healthy. We group the infected cells according to the way they were infected and obtained the system of Eqs (26) and (27). Numerical simulation for the proportions of cells infected per time step grouped according to the mode of viral spread are given in Fig 9. It can be seen that both forms of viral spread result in new cells being infected, however, the relative contribution of cell-associated viral spread is higher than that of cell-free viral spread with a marked distinction in the first few days of the infection.

**Fig 10:**
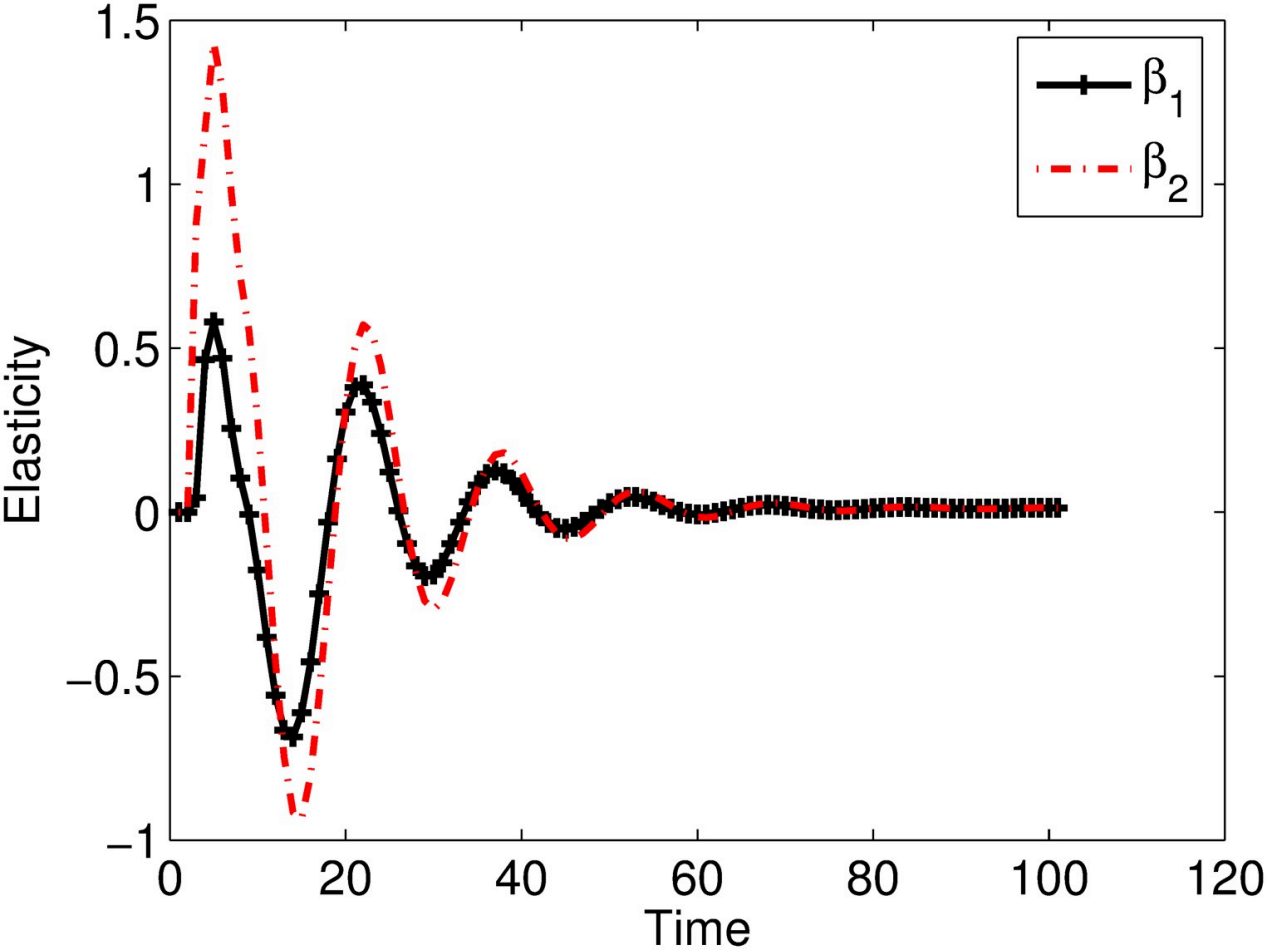
Plot of the elasticities of the viral levels to the transmission parameters. The viral level is more elastic to *β*_2_ than *β*_1_.

Elasticity analysis of the viral levels to transmission parameters *β*_1_ and *β*_2_ are given in Fig 10. It is shown that during the early days of the infection the viral level is more elastic to *β*_2_ than *β*_1_, a result similar to the above analysis that showed that cell-associated viral spread is more efficient in spreading the infection than cell-free viral spread.

Elasticity analysis also predicts that viral levels are only sensitive to the transmission parameters during the early days of the infection. After infection establishment, strategies that target other stages of the viral life cycle can control viral levels better than those targeting the early stages of viral life cycle. These results are similar to the results obtained in [36–39].

## Discussion

HIV infection is currently treated by highly active antiretroviral therapy (HAART), a therapy that only slows the development of AIDS in an infected individual and there is need to find a cure or a vaccine that prevents the occurrence of new infections. The development of a vaccine or a microbicide that prevents the occurence of new infection heavily relies on understanding the form of the virus that spreads efficiently. Even so, the form of HIV that spread efficiently is not yet clear [4, 40, 41]. Currently most of the vaccine research concentrate on cell-free viral spread [4, 40], though there are indications that cell-associated virus may play a significant role in the transmission of the HIV infection [16, 17, 20]. Discrete time models which consider the two forms of HIV spread across target cells were developed with the goal of determining the form of viral spread that is more efficient between cell-free and cell-associated viral spread.

The work started by developing a model that considered cell-free viral spread. The qualitative behavior of the model showed that the disease free equilibrium was the only stable equilibrium point for biologically feasible parameter sets. A result that implies that infection through cell-free viral spread will die out. A model that considered cell-associated viral spread was also developed. Existence of the disease equilibrium was shown to depend on the cell-associated transmission parameter. For small values of the cell-associated transmission parameter, the disease free equilibrium is stable and disease equilibrium is stable for parameter values greater than 0.005. From these results, we conclude that cell-free viral spread fails to thrive because of the low infectivity of cell-free virus and cell-associated viral spread thrives because of the high infectivity of cell-associated virus.

Since the infectious fluids contains both cell-free and cell-associated virus, a model that considers both forms of viral spread was also formulated. Plots of the proportions of cells infected by cell-free virus and cell-associated virus per time step showed that the proportion of cells infected by cell-associated virus was greater than that infected by cell-free virus with a marked difference during the early days of the infection. This result implies that the relative contribution of cell-associated virus in spreading the infection is greater than that of the cell-free virus. Elasticity analysis of the viral levels to the transmission parameters was performed. It was shown that the viral levels (and hence the infection) was more elastic to the infectivity of the cell-associated virus than the infectivity of free virus during the first few days of the infection. A result that also implies that the relative contribution of cell-associated virus in spreading HIV from primary sites of infection is greater than that of cell-free virus.

Our results coincide with the result in [19], where it was shown that a newly infected homosexual man was infected by the virus that was closely related to the transmitting partner’s seminal cells and not the seminal plasma virus and another study [17], where the cell-associated viral levels correlated with the risk of acquisition. In the study [10], the genotype of infecting virus matched that of HIV in semen cells of the transmitter in three (one heterosexual couple) out of five cases, a result that also suggested that cell-associated virus is a major contributor of new infections in humans. There are also *ex vivo* and *in vivo* model results that coincide with our result see for example [41].

Our models produces different results for different stages of the infection. We have shown that upon exposure to the virus, cell-associated viral spread is more efficient in spreading the virus, however with infection progression, two forms of viral spread contribute almost equally. Our results therefore agree with the results of the study [30] which concluded that the two modes of viral spread contribute equally to viral spread but with a difference that our models predict a marked difference in the early days of the infection. The difference maybe because of our use of matrix population models that can easily capture both the transient and asymptotic dynamics of the viral levels.

Our results also coincide with results of the study [31], which also used a mathematical model to quantify the contribution of the two forms of viral spread and concluded that cell-associated viral spread is more efficient than cell-free viral spread. We postulate that the differences in the results obtained in different studies could have been the timing of the experiments, some researchers could have been studying the transient dynamics hence obtaining a result that cell-associated virus is more efficient in spreading the infection than cell-free virus and others could have looked at the stage were the virus had established itself, where they would get the result that the two forms of viral spread contribute almost equally.

In this study, intervention strategies that target cell-associated viral spread are predicted to be more beneficial in controlling the spread of the HIV infection than those that target cell-free viral spread. Given the differences in which these two forms of transmission occur, vaccines and microbicides designed to prevent cell-free viral spread may not control cell-associated viral spread [13, 42]. This could explain why some vaccines and clinical trials designed to prevent HIV infection may have failed. Cell-to-cell viral spread can be controlled by microbicides of the surfactant class that disrupt membranes of infectious cells in genital secretions and semen, buffering agents that induce lower vaginal pH after intercourse and antibodies that target infected cells, just to mention a few [40].

There are gaps in knowledge on what transpires in continuously shaken cultures. Microscopic and micro-imaging techniques can improve our knowledge on what really transpires in continuously shaken cultures. Once the kinetics that occur in shaken cultures are clearly elucidated, the use of cell cultures in the estimation of parameters on models that consider the different forms of viral spread will help us correctly quantify the contribution of each form of viral spread.

Moreover, most active HIV replication occurs in lymph nodes [43, 44] where about 98% of lymphocytes reside [45, 46]. Understanding the mode of viral spread more efficient in these regions is of paramount importance in the control of the infection. However, cells are closely packed in lymphoid organs and are in the ranges of 10^6^ cells per microliter and the dynamics of cells and the virus in lymphoid organs cannot be modelled with the models proposed in this study, where we assumed perfect mixing of cells and the virus. Cellular automata models can be used to model the dynamics in these regions and inform on the role of each form of viral spread in the HIV infection dynamics.

## Models

The stages in the HIV replication cycle are; receptor binding, cell entry, uncoating, reverse transcription of viral RNA into DNA, nuclear entry, integration of the viral DNA into the host DNA and transcription and translation of viral RNA, assembly of progeny virus particles and budding [47]. The life cycle stages of the virus are divided into three stages: the mature virus (*V*), the HIV Deoxyribonucleic Acid (DNA) (*D*) and the Provirus (*P*) as illustrated in our previous study [48]. A Negative Binomial distribution method is used to estimate the duration of the provirus stage. This resulted in two pseudo-provirus stages as illustrated in [48]. We let *P* represent the pseudo-provirus stage 1 and *Q* represent the pseudo-provirus stage 2.

### A discrete time cell-free viral spread model

This model considers cell-free viral spread whereby health cells are infected through contacts with free virus particles. We assume that a CD4^+^ T cell is infected by free virus particles through contacts of uninfected cells with the virus in a random fashion (contacts are assumed to be randomly distributed). Thus the model represent cell and virus interactions in the blood where perfect mixing of the cells and the virus is assumed. We let *D*_*t*_, *P*_*t*_, *Q*_*t*_, *V*_*t*_, *T*_*t*_ and $T_{t}^{*}$ be the densities of the DNA, provirus stage 1, provirus stage 2, virus, the uninfected and infected CD4^+^ T cells per ml of blood at time *t*, respectively. Adopting the principles used in host-parasite interactions for the processes of attachment and entry of the virus into CD4^+^ T cells, we assume that the infection of CD4^+^ T cells is determined by the number of contacts/encounters of the virus with the CD4^+^ T cells. The number of contacts are assumed to be proportional to the product of the densities of the virus and the CD4^+^ T cells, that is *N* = *β*_1_*V*_*t*_*T*_*t*_, where *β*_1_ is the constant of proportionality representing the infectivity of the virus. The first contact is the only significant encounter. Since the encounters are assumed to be random, the encounters are described by a Poisson probability distribution and the average number of contacts per host (average number of virus attached to a CD4^+^ T cell) is given by *β*_1_*V*_*t*_. Since the likelihood of escaping infection is the same as the probability of no encounters during the CD4^+^ T cell’s lifetime, the proportion of cells that survive infection per time step is given by the probability of zero encounters as *P*(0) = exp(−*β*_1_*V*_*t*_). The HIV cell-free infection model takes the form:

$$\begin{array}{ccc} D_{t+1} & = & \beta_{1}V_{t}, \end{array}$$ (1)

$$\begin{array}{ccc} P_{t+1} & = & \theta_{1}D_{t}+\theta_{3}P_{t}, \end{array}$$ (2)

$$\begin{array}{ccc} Q_{t+1} & = & \theta_{2}P_{t}+\theta_{3}Q_{t}, \end{array}$$ (3)

$$\begin{array}{ccc} V_{t+1} & = & \theta_{2}\phi Q_{t}T_{t}^{*}, \end{array}$$ (4)

$$\begin{array}{ccc} T_{t+1} & = & s_{T}+\gamma T_{t}\exp(-\frac{T_{t}+T_{t}^{*}}{K}-\beta_{1}V_{t}), \end{array}$$ (5)

$$\begin{array}{ccc} T_{t+1}^{*} & = & T_{t}\left[1-\exp\left(-\beta_{1}V_{t}\right)\right]+\left(1-\mu \right)T_{t}^{*}, \end{array}$$ (6)

where *β*_1_, *θ*_1_, *θ*_2_, *θ*_3_, *ϕ*, *γ*, *K*, *μ* and *s*_*T*_ are positive parameters and *D*_0_, *P*_0_, *Q*_0_, *V*_0_, *T*_0_, * *T*_0_ ≥ 0. The time step for the model is 12 hours, which is equivalent to the amount of time spend in the *D* stage. Eq (1) models the amount of the DNA particles in a cell at time *t* + 1. Not all viruses that attach to a cell are successfully fused with the cell, uncoated and reverse transcribed [49, 50]. This means that *β*_1_, is a product of the probabilities of attachment, fusion, uncoating and reverse transcription.

It has been observed that all HIV unintegrated DNA are rapidly transported to the nucleus where they are either processed into the two types of DNA circles or integrated [51]. The DNA particles that circularise do not contribute to infection progression and are eventually degraded by the cell. This means that only those DNA particles that become integrated are involved in the infection dynamics and those that circularise though they maybe available in the next time step no longer participate in the infection dynamics. Thus if *D* does not integrate and become *P* at time *t*, it is assumed that it will not be available at time *t* + 1.

Provirus particles progress from provirus stage to the mature virus stage over a variable time. To calculate the proportion that goes to the virus stage and the proportion that remains in the provirus stage we used the Negative Binomial Distribution method as illustrated in [48]. The approach resulted in two identical pseudo provirus stages which we denote by *P* and *Q*. Eq (2) represents the amount of provirus in the pseudo provirus stage 1 and Eq (3) represents the amount of provirus in the pseudo provirus stage 2 at time *t* + 1 respectively. The parameter *θ*_2_, is the transition probability within these stages and between the provirus stage and the virus stage. The probability that a provirus survives and remain in the same pseudo provirus stage is given by *θ*_3_.

It has been assumed that the viral level at time *t* + 1, does not depend on the viral level at time *t*, because plasma virus have a mean life span of 0.3 days [21] and the time step for the model is 0.5 days, meaning that no plasma virus is able to survive to the next time step. The parameter *ϕ* represents the number of virus particles produced per replication cycle per infected cell. The expression *θ*_2_*ϕQ*_*t*_ gives virus production per cell. To get the virus production per ml of blood we multiply by the number of infected cells, T*, per ml of blood.

Eq (5) models the levels of CD4^+^ T cells at time *t* + 1. The parameter *s*_*T*_, represents the source of new CD4^+^ T cells from the thymus. It is assumed that addition from the thymus occurs at the beginning of the time step. We have assumed a density dependent growth function for these cells of the form $\gamma T_{t}\exp(-\frac{T_{t}+T_{t}^{*}}{K})$. This was motivated by the fact that CD4^+^ T levels are regulated in vivo. Exponential functions are used when the rate of change of a substance depends on the current level, in this case the level of health cells at time *t* + 1 depends on the levels of both the infected and uninfected cells at time *t* and these cells have a carrying capacity of K cells per *ml* of blood. The exponential term therefore regulates the levels per each time step.

To proceed to the next time step, CD4^+^ T cells must survive infection by free virus. Since the likelihood of escaping infection is the same as the probability of no contacts during the CD4^+^ T cell’s lifetime, the probability of the CD4^+^ T surviving infection is given by exp(−*β*_1_*V*_*t*_). Infection is assumed to occur at the end of the time step so that the expression, $\gamma T_{t}\exp(-\frac{T_{t}+T_{t}^{*}}{K}-\beta_{1}V_{t})$, gives the number of CD4^+^ T cells that survive infection per time step. The expression (1 − exp(−*β*_1_*V*_*t*_)), gives the proportion of cells that become infected per time step, so that *T*_*t*_(1 − exp(−*β*_1_*V*_*t*_)) gives the gain term per time step. The parameter *μ*, gives the proportion of infected cells that die per time step, and 1 − *μ*, gives the proportion that survive per time step. Death is assumed to occur at the end of each time step.

Eqs (1)–(3), give the intracellular stages of the virus and the densities give the levels per infected cell. To get the levels per ml of blood we multiply by the total number of infected cell per ml of blood, $T_{t}^{*}$. The distribution of the virus population at the different life cycle stages at time *t* + 1 can be represented in matrix form for easier manipulations as follows,

$$\begin{array}{c} N(t+1)=AN(t). \end{array}$$ (7)

where $N\left(t\right)={\left(\begin{array}{cccc} D_{t}, & P_{t}, & Q_{t}, & V_{t} \end{array}\right)}^{\prime },\;A=(\begin{array}{cccc} 0 & 0 & 0 & \beta_{1}\\ \theta_{1} & \theta_{3} & 0 & 0\\ 0 & \theta_{2} & \theta_{3} & 0\\ 0 & 0 & \theta_{2}\phi T_{t}^{*} & 0 \end{array})$ and the equations for uninfected and infected cells remain as previously defined. The equilibrium value of infected cells, T* is given by

$$\begin{array}{c} s_{T}-T^{*}+\gamma (T^{*}-\mu T^{**})\exp\left(-\frac{T^{*}+T^{**}}{K}\right)=0. \end{array}$$ (8)

**Fig 11:**
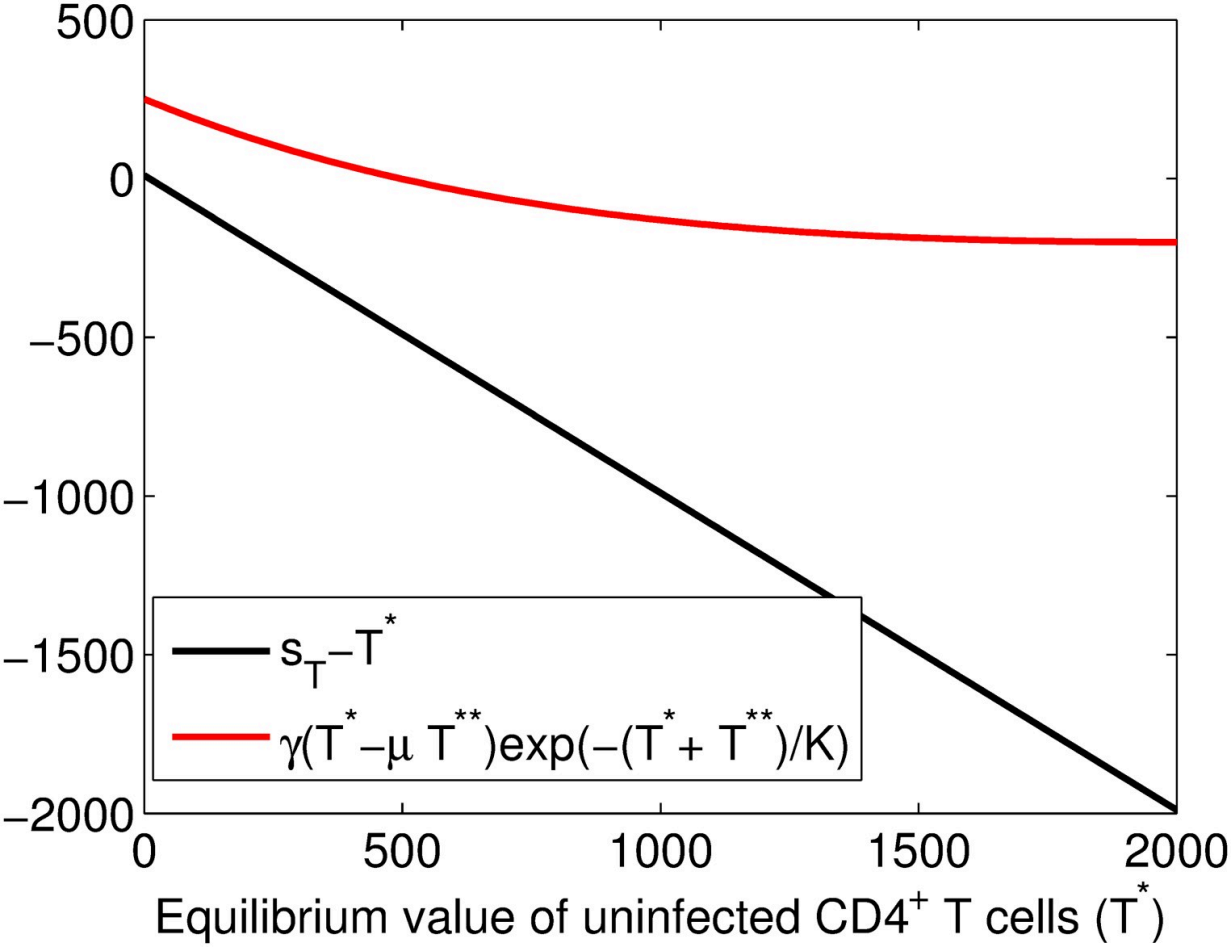
Plots of *s*_*T*_ − T* and $-\gamma (T^{*}-\mu T^{**})\exp\left(-\frac{T^{*}}{K}\right)$ against T*. The graphs are not intersecting and thus there is no solution for the equation. The parameters used were $\theta_{1}=\frac{1}{3}$, *θ*_2_ = 0.06315, *θ*_3_ = 0.43685, *β*_1_ = 0.000024, *ϕ* = 1000, *s_T_* = 10, *μ* = 0.5, *K* = 1500, *γ* = 2.7.

Solving Eq (8) is challenging and we resort to the graphical method. The equation is written as

$$\begin{array}{c} T^{*}-s_{T}=\gamma (T^{*}-\mu T^{**})\exp\left(-\frac{T^{*}+T^{**}}{K}\right), \end{array}$$ (9)

where $T^{**}=\frac{{\left(1-\theta_{3}\right)}^{2}}{\theta_{1}\theta_{2}^{2}\phi \beta_{1}}$. We plot the equation *s*_*T*_ − T* on the same plot with $-\gamma (T^{*}-\mu T^{**})\exp\left(-\frac{T^{*}+T^{**}}{K}\right)$, where their graphs intersect is the solution of Eq (8) and the plots are given in Fig 11. The graphs are not intersecting and thus there is no solution for the given parameter set.

In order to determine if the transmission parameter *β*_1_, can contribute to the qualitative behavior of the model, we plot a bifurcation diagram of the cell levels against the transmission parameter Fig 1. Model analysis and the derivations of equilibrium values are given in the supporting information.

### A discrete time cell-associated viral spread model

Immune cells communicate through short- and long-range connections. HIV takes advantage of these connections to disseminate through cell-to-cell viral spread. A review of how cell-to-cell viral spread occurs is given in [40]. Cell-to-cell spread of HIV depends on the formation of a virological synapse. We thus assume that infection of CD4^+^ T cells is determined by the number of contacts/encounters of infected cells and health CD4^+^ T cells. The model explains interactions of cells in the blood compartment where random mixing of cells can be assumed. The cell-associated viral spread model takes the form

$$\begin{array}{ccc} D_{t+1} & = & \beta_{2}T_{t}^{*}, \end{array}$$ (10)

$$\begin{array}{ccc} P_{t+1} & = & \theta_{1}D_{t}+\theta_{3}P_{t}, \end{array}$$ (11)

$$\begin{array}{ccc} Q_{t+1} & = & \theta_{2}P_{t}+\theta_{3}Q_{t}, \end{array}$$ (12)

$$\begin{array}{ccc} V_{t+1} & = & \theta_{2}\phi Q_{t}T_{t}^{*}, \end{array}$$ (13)

$$\begin{array}{ccc} T_{t+1} & = & s_{T}+\gamma T_{t}\exp(-\beta_{2}T_{t}^{*}-\frac{T_{t}+T_{t}^{*}}{K}), \end{array}$$ (14)

$$\begin{array}{ccc} T_{t+1}^{*} & = & T_{t}(1-\exp\left(-\beta_{2}T_{t}^{*}\right))+\left(1-\mu \right)T_{t}^{*}. \end{array}$$ (15)

The expression $\beta_{2}T_{t}^{*}$ gives the average number of infected cell contacts per health CD4^+^ T cell (average number of infected cells attached to a health cell), where *β*_2_ represents the infectivity of cell-associated virus. We have assumed that all the virus that become attached to the cell will enter the cell as with the cell-free model. All the other parameters and variables are as previously defined in the cell-free viral spread model.

The system of Eqs (10)–(15) can be represented in matrix form for easier manipulation as,

$$\begin{array}{ccc} N(t+1) & = & GN(t)+Bn(t), \end{array}$$ (16)

$$\begin{array}{ccc} n(t+1) & = & En(t)+F, \end{array}$$ (17)

where $N(t)={\left(\begin{array}{cccc} D_{t} & P_{t} & Q_{t} & V_{t} \end{array}\right)}^{\prime },n(t)={\left(\begin{array}{cc} T_{t} & T_{t}^{*} \end{array}\right)}^{\prime },B=\left(\begin{array}{cc} 0 & \beta_{2}\\ 0 & 0\\ 0 & 0\\ 0 & 0 \end{array}\right),$

$$F={\left(\begin{array}{cc} s_{T} & 0 \end{array}\right)}^{\prime },\;\;\;E=(\begin{array}{cc} \gamma \exp(-\beta_{2}T_{t}^{*}-\frac{T_{t}+T_{t}^{*}}{K}) & 0\\ \left(1-\exp\left(-\beta_{2}T_{t}^{*}\right)\right) & 1-\mu \end{array})$$

and

$$G=(\begin{array}{cccc} 0 & 0 & 0 & 0\\ \theta_{1} & \theta_{3} & 0 & 0\\ 0 & \theta_{2} & \theta_{3} & 0\\ 0 & 0 & \theta_{2}\phi T_{t}^{*} & 0 \end{array}).$$

The equilibrium value of infected cells is given by

$$\begin{array}{c} f\left(T^{**}\right)\mu T^{**}-s_{T}\left(1-\exp\left(-\beta_{2}T^{**}\right)\right)-\gamma \mu T^{**}\exp(-\beta_{2}T^{**}-\frac{\mu T^{**}}{K(1-\exp\left(-\beta_{2}T^{**}\right))}-\frac{T^{**}}{K})=0. \end{array}$$ (18)

Solving for T** is complex and we resort to graphical solutions. We let

$$\begin{array}{c} f\left(T^{**}\right)=\mu T^{**}-s_{T}\left(1-\exp\left(-\beta_{2}T^{**}\right)\right)-\gamma \mu T^{**}\exp(-\beta_{2}T^{**}-\frac{\mu T^{**}}{K(1-\exp\left(-\beta_{2}T^{**}\right))}-\frac{T^{**}}{K}). \end{array}$$ (19)

The graphs of *f*(T**) against T** are given in Fig 5. Model analysis and the derivations of equilibrium values are given in the supporting information.

### Cell-free and cell-associated viral spread model

The model that considers both forms transmission takes the form;

$$\begin{array}{ccc} D_{t+1} & = & \beta_{1}V_{t}+\beta_{2}T_{t}^{*}, \end{array}$$ (20)

$$\begin{array}{ccc} P_{t+1} & = & \theta_{1}D_{t}+\theta_{3}P_{t}, \end{array}$$ (21)

$$\begin{array}{ccc} Q_{t+1} & = & \theta_{2}P_{t}+\theta_{3}Q_{t}, \end{array}$$ (22)

$$\begin{array}{ccc} V_{t+1} & = & \theta_{2}\phi Q_{t}T_{t}^{*}, \end{array}$$ (23)

$$\begin{array}{ccc} T_{t+1} & = & s_{T}+\gamma T_{t}\exp(-\beta_{1}V_{t}-\beta_{2}T_{t}^{*}-\frac{T_{t}+T_{t}^{*}}{K}), \end{array}$$ (24)

$$\begin{array}{ccc} T_{t+1}^{*} & = & T_{t}(1-\exp\left(-\beta_{1}V_{t}-\beta_{2}T_{t}^{*}\right))+\left(1-\mu \right)T_{t}^{*}. \end{array}$$ (25)

The definitions of parameters and variables remain as previously defined. In this model, it is assumed that a cell must survive infection by infected cells and cell free viral particles for it to remain healthy. Boundedness of the system of Eqs (20)–(25) follows from the boundedness of system of Eqs (1)–(6) and (10)–(15).

#### Contributions of the two forms of viral spread

We group the infected cells according to the way they were infected and the resulting equations for infected cells are

$$\begin{array}{ccc} T1_{t+1}^{*} & = & \frac{\beta_{1}V_{t}}{\beta_{1}V_{t}+\beta_{2}T_{t}^{*}}T_{t}(1-\exp\left(-\beta_{1}V_{t}-\beta_{2}T_{t}^{*}\right))+\left(1-\mu \right)T1_{t}^{*} \end{array}$$ (26)

$$\begin{array}{ccc} T2_{t+1}^{*} & = & \frac{\beta_{2}T_{t}^{*}}{\beta_{1}V_{t}+\beta_{2}T_{t}^{*}}T_{t}(1-\exp\left(-\beta_{1}V_{t}-\beta_{2}T_{t}^{*}\right))+\left(1-\mu \right)T2_{t}^{*}, \end{array}$$ (27)

where $T_{t}^{*}=T1_{t}^{*}+T2_{t}^{*}$, $T1_{t}^{*}$ are cells infected by free virus and $T2_{t}^{*}$ are cells infected by cell-associated virus at time *t* respectively. Numerical simulation for the proportions of cells infected per time step grouped according to the mode of transmission are given in Fig 9. Both forms of viral spread result in new cells being infected, however, the relative contribution of cell-associated viral spread is higher than that of cell-free viral spread.

The mathematical analysis of all the models are given in the supporting information.

### Model parameters

In [51], the investigators revealed that all HIV unintegrated DNA are rapidly transported to the nucleus where they are either processed into the two types of DNA circles or integrated. It was observed that only a third will become integrated. We can thus approximate the proportion of DNA (D) that goes to the provirus stage as $\frac{1}{3}$ that is $\theta_{1}=\frac{1}{3}$. The stage specific survival in the provirus stage is the proportion of replication competent provirus and was determined as $\frac{1}{2}$ [52]. The provirus stage take a variable duration, we used the Negative Binomial Distribution method to estimate its duration as illustrated in one of our studies [48]. This resulted in two pseudo-provirus stages with transition probability *θ*_2_ = 0.06315 and the probability of surviving and remaining in the same stage *θ*_3_ = 0.43685. The parameters of the model are given in Table 1.

**Table 1:** Parameter values.

Parameter	Description	Value	Source
*s*_*T*_	Source terms of CD4^+^ T cells from thymus	10*cellsd*^−1^	[48]
K	Carrying capacity of CD4^+^ T cells	1500 cells ml^−1^	Est.
*μ*	Proportion of infected cells that die per time step	0.5 to 1	[33]
*γ*	Growth rate of CD4^+^ T cells	2.7 cells d^−1^	Est.
*β*_1_	Virus infectivity	2.4 × 10^−5^ *ml*^−1^ *d*^−1^	[33]
*β*_2_	Cell-associated virus infectivity	100 − 1000 × *β*_1_	[6, 8, 53]
*ϕ*	Virus production/provirus/cycle	1000 virions	[29]
*θ*_1_	Transition Probability	$\frac{1}{3}$	[33]
*θ*_2_	Transition Probability	0.06315	[48]
*θ*_3_	Probability of surviving and remaining in provirus stages	0.43685	[48]

Est. means that parameters were estimated/derived to simulate acceptable HIV dynamics.

## Supporting information

S1 File Appendix. Mathematical analysis of the models.
(PDF)
