# Supplementary material for: On the efficiency of HIV transmission: Insights through discrete time HIV models
Source: PLoS One. 2019 Sep 18;14(9):e0222574. doi: 10.1371/journal.pone.0222574 (PMC6750597; doi:10.1371/journal.pone.0222574)
Supplement: S1 File — Mathematical analysis of the models. (PDF) [file pone.0222574.s001.pdf]

On the efficiency of HIV transmission: Insights through discrete time HIV models

Showa SP, Nyabadza F. and Hove-Musekwa SD.

Appendix

Basic properties of the cell free viral spread model

The cell-free viral spread model is given by the following system of equations;

$$\begin{aligned} D_{t+1} &= \beta_1 V_t, & (1)\\ P_{t+1} &= \theta_1 D_t + \theta_3 P_t, & (2)\\ Q_{t+1} &= \theta_2 P_t + \theta_3 Q_t, & (3)\\ V_{t+1} &= \theta_2 \phi Q_t T_t^*, & (4)\\ T_{t+1} &= s_T + \gamma T_t \exp\left(-\frac{T_t + T_t^*}{K} - \beta_1 V_t\right), & (5)\\ T_{t+1}^* &= T_t[1 - \exp(-\beta_1 V_t)] + (1-\mu) T_t^*, & (6) \end{aligned}$$

The system of equations (1)-(6) will be studied in a biologically feasible region. We begin by showing that the solutions of this system of equations are bounded.

Proposition 1. *The system of equations (1)-(6) is bounded.*

Proof. For any $t_0 \in \mathbb{Z}^+$, eq (5), gives

$$\begin{aligned} T_{t_0+1} &\leq s_T + \gamma T_{t_0} \exp\left(-\frac{T_{t_0}}{K}\right), & (7)\\ &\leq s_T + \gamma K \exp(-1), \end{aligned}$$

where we have used $\max_{x\in\mathbb{R}} x \exp(-x/K) = K \exp(-1)$. We claim that

$$T_t \leq s_T + \gamma K \exp(-1) \quad \text{for t} \geq \text{t}_0.$$

We prove this by contradiction. Assume that there exist a number $\bar{l}_0 > t_0$ such that $T_{\bar{l}_0} > s_T + \gamma K \exp(-1)$. Then $\bar{l}_0 \geq t_0 + 2$. From the previous argument we have

$$\begin{aligned} T_{\bar{l}_0} &\leq s_T + \gamma T_{\bar{l}_0 - 1} \exp\left(-\frac{T_{\bar{l}_0-1}}{K}\right), & (8)\\ &\leq s_T + \gamma K \exp(-1), \end{aligned}$$

which is a contradiction.

From eq (6), it can be deduced that,

$$\begin{aligned} T^*_{t+1} &\leq T_t + (1-\mu)T^*_t \\ &\leq s_T + \gamma K \exp(-1) + (1-\mu)T^*_t. \end{aligned}$$

The solution of $T^*_{t+1} = s_T + \gamma K \exp(-1) + (1-\mu)T^*_t$ is given by

$$T^*_t = (1-\mu)^t T^*_0 + (s_T + \gamma K \exp(-1)) \sum_{i=0}^{t-1} (1-\mu)^i.$$

However, $\sum_{i=0}^{t-1}(1-\mu)^i$ is a geometric series such that

$$T^*_t = \begin{cases} (1-\mu)^t T^*_0 + (s_T + \gamma K \exp(-1)) \left(\frac{1-(1-\gamma_{T^*})^t}{\gamma_{T^*}}\right) & \text{if } (1-\mu) \neq 1, \\ T^*_0 + (s_T + \gamma K \exp(-1))t & \text{if } (1-\mu) = 1. \end{cases}$$

The parameter $\mu \neq 0$ and thus we have

$$T^*_t \leq (1-\gamma_{T^*})^t T^*_0 + (s_T + \gamma K \exp(-1)) \left(\frac{1-(1-\mu_{T^*})^t}{\mu_{T^*}}\right).$$

In this case

$$\limsup_{t\to\infty} T^*_t \leq \frac{s_T + \gamma K \exp(-1)}{\mu_{T^*}}.$$

If we let

$$n(t) = \begin{pmatrix} T_t & T^*_t \end{pmatrix}' \text{ and } \hat{\text{n}} = \begin{pmatrix} s_T + \gamma K \exp(-1) & \frac{s_T + \gamma K \exp(-1)}{\mu_{T^*}} \end{pmatrix}',$$

then

$$n(t) \leq \hat{n} \text{ for all t.}$$

Observe that

$$N(t+1) \leq \hat{A} N(t), \tag{9}$$

where

$$\hat{A} = \begin{pmatrix} 0 & 0 & 0 & \beta_1 \\ \theta_1 & \theta_3 & 0 & 0 \\ 0 & \theta_2 & \theta_3 & 0 \\ 0 & 0 & \theta_2 \phi \frac{s_T + \gamma K \exp(-1)}{\mu_{T^*}} & 0 \end{pmatrix}.$$

Define

$$N_1(t+1) = \hat{A} N_1(t), \quad t > 0, \quad N_1(0) = \hat{A} N(0),$$

then

$$N_1(t) = \hat{A}^t N_1(0).$$

The spectral radius of $\hat{A}$, is less than 1, and thus $N_1(t)$, is decreasing sequence. This means that $\hat{A}^t N_1(0) \to \mathbf{0}$, as $t \to \infty$, where $\mathbf{0} = \begin{pmatrix} 0 & 0 & 0 & 0 \end{pmatrix}$. We therefore get that

$$\liminf_{t\to\infty} N_1(t) \geq \mathbf{0} \tag{10}$$

From equality (9) and (10), it can be seen that $\mathbf{0} \leq N(t) \leq N_1(0)$. □

We thus have the following proposition.

Proposition 2. *The set defined by*

$$\Omega = \{N(t), n(t) \in \mathbb{R}^4_+ \times \mathbb{R}^2_+ : \mathbf{0} \leq N(t) \leq N_1(0),\ \bar{\mathbf{0}} \leq n(t) \leq \hat{n}\},$$

where $\bar{\mathbf{0}} = \begin{pmatrix} 0 & 0 \end{pmatrix}$, *is positively invariant under the flows of the system of equations (1)-(6).*

Fixed points

The equilibrium points (fixed points) are obtained by equating the right hand side to the left hand side of the system of equations (1)-(6). If we denote the equilibrium point by $\begin{pmatrix} D^*, & P^*, & Q^*, & V^*, & T^*, & T^{**} \end{pmatrix}'$, we have the following system of equations

$$\begin{aligned}
D^* &= \beta_1 V^*, & (11)\\
P^* &= \theta_1 D^* + \theta_3 P^*, & (12)\\
Q^* &= \theta_2 P^* + \theta_3 Q^*, & (13)\\
V^* &= \theta_2 \phi Q^* T^{**}, & (14)\\
T^* &= s_T + T^* \gamma \exp(-\beta_1 V^* - \frac{T^* + T^{**}}{K}), & (15)\\
T^{**} &= T^*(1 - \exp(-\beta_1 V^*)) + (1-\mu) T^{**}. & (16)
\end{aligned}$$

Substituting eq (11) into eq (12) we have

$$P^* = \frac{\theta_1}{1-\theta_3}(\beta_1 V^*). \tag{17}$$

Eq (17) into eq (13) result in

$$Q^* = \frac{\theta_1 \theta_2}{(1-\theta_3)^2}(\beta_1 V^*). \tag{18}$$

Substituting eq (18) into eq (14) we have

$$V^* = \frac{\theta_1 \theta_2^2 \phi}{(1-\theta_3)^2}(\beta_1 V^*) T^{**}. \tag{19}$$

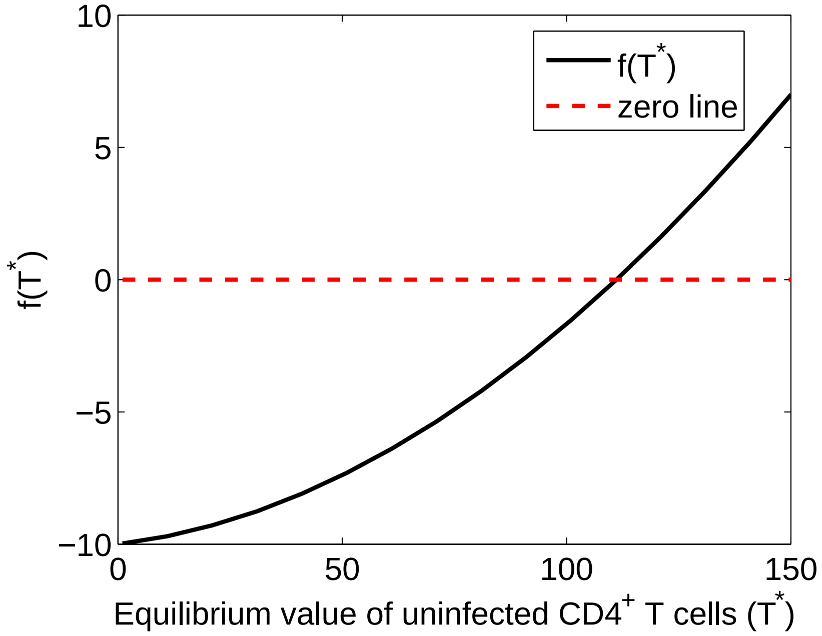

Figure 1: A graph of $f(T^*)$ against T^*. The parameters used were $s_T = 10,\ K = 1500,\ \gamma = 2.7$. It can be seen that the graph of $f(T^*)$ crosses the x-axis and thus there is solution for this parameter set.

From eq (19), we get $V^* = 0$, which gives the disease free equilibrium point or $T^{**} = \frac{(1-\theta_3)^2}{\theta_1\theta_2^2\phi\beta_1}$. Substituting $V^* = 0$ into equations (11)-(16) we get the disease free equilibrium

$$E_0 = \begin{pmatrix} 0, & 0, & 0, & 0, & T^*, & 0 \end{pmatrix}',$$

where T^* is a solution of

$$T^* - s_T - T^*\gamma \exp{(-\frac{T^*}{K})} = 0. \tag{20}$$

The analytical solution to the eq (20) is complex therefore, the graphical method is used to show the existence of the solution. Let $f(T^*) = T^* - s_T - T^*\gamma \exp{(-\frac{T^*}{K})}$, then the graph of $f(T^*)$ against T^* is given in Figure 1. It can be seen from Figure 1 that the graph of $f(T^*)$ crosses the x-axis and thus there is a solution to the eq (20) for this parameter set.

From eq (16) we have

$$1-\frac{\mu T^{**}}{T^{*}}=\exp(-\beta_1 V^{*}). \tag{21}$$

Substituting eq (21) into (15) result in

$$s_T-T^{*}+T^{*}\gamma\left(1-\frac{\mu T^{**}}{T^{*}}\right)\exp\,(-\frac{T^{*}}{K})=0, \tag{22}$$

which can be simplified to give

$$s_T-T^{*}+\gamma\,(T^{*}-\mu T^{**})\exp\,(-\frac{T^{*}+T^{**}}{K})=0, \tag{23}$$

where $T^{**}=\frac{(1-\theta_3)^2}{\theta_1\theta_2^2\phi\beta_1}$.

Stability of fixed points

The following theorem in [1] is used to study stability of the equilibrium points.

Theorem 1. *A fixed point x^* of a function $f(x)$ is asymptotically stable if all the eigenvalues μ of the first derivative, $Df(x)$ of $f(x)$ at x^* satisfy $|\mu|<1$. The fixed point x^* is unstable if there exists an eigenvalue μ such that $|\mu|>1$.*

Proposition 3. *The disease free equilibrium point*

$$E_0=\begin{pmatrix} 0, & 0, & 0, & 0, & T^{*}, & 0\end{pmatrix}',$$

exist and is locally asymptotically stable when

$$\left|\gamma\left(1-\frac{T^{*}}{K}\right)\exp\left(-\frac{T^{*}}{K}\right)\right|<1,$$

where T^* is the solution of eq (20).

Proof. Existence has been proved in the previous section. The Jacobian matrix at this equilibrium is given by

$$J_0=\begin{pmatrix} 0 & 0 & 0 & \beta_1 & 0 & 0\\ \theta_1 & \theta_3 & 0 & 0 & 0 & 0\\ 0 & \theta_2 & \theta_3 & 0 & 0 & 0\\ 0 & 0 & 0 & 0 & 0 & 0\\ 0 & 0 & 0 & M & G1 & 0\\ 0 & 0 & 0 & -\beta_1 T^{*} & 0 & 1-\mu \end{pmatrix},$$

where

$$M = -\beta_1 \gamma T^* \exp\left(-\frac{T^*}{K}\right),$$

and

$$G1 = \gamma \left(1 - \frac{T^*}{K}\right) \exp\left(-\frac{T^*}{K}\right).$$

All the eigenvalues J_0 (0 twice, θ_3 twice, G1 and $1-\mu$) have magnitudes less than one if $|G1| < 1$. This completes the proof. $\square$

Proposition 4. *The system of equations (1)-(6), does not have an endermic equilibrium point for biologically feasible parameters.*

Basic properties of the cell-associated viral spread model

The cell-associated viral spread model is given by the following system of equations;

$$\begin{aligned}
D_{t+1} &= \beta_2 T_t^*, && (24)\\
P_{t+1} &= \theta_1 D_t + \theta_3 P_t, && (25)\\
Q_{t+1} &= \theta_2 P_t + \theta_3 Q_t, && (26)\\
V_{t+1} &= \theta_2 \phi Q_t T_t^*, && (27)\\
T_{t+1} &= s_T + \gamma T_t \exp\left(-\beta_2 T_t^* - \frac{T_t + T_t^*}{K}\right), && (28)\\
T_{t+1}^* &= T_t \left(1 - \exp\left(-\beta_2 T_t^*\right)\right) + (1-\mu) T_t^*. && (29)
\end{aligned}$$

Proposition 5. *The solutions of the system of equations (24)-(29) remain non negative and are bounded. The set* Θ *defined by* $\Theta = \{N(t), n(t)) \in \mathbb{R}^4 \times \mathbb{R}^2 : \boldsymbol{0} \leq N(t) \leq (I - \bar{G})^{-1} B\bar{n}, \bar{\boldsymbol{0}} \leq n(t) \leq \bar{n}\}$*, where* $\bar{n} = \begin{pmatrix} s_T + \gamma K \exp(-1) & \frac{s_T + \gamma K \exp(-1)}{\mu} \end{pmatrix}'$, $\boldsymbol{0} = \begin{pmatrix} 0, & 0, & 0, & 0 \end{pmatrix}'$, $\bar{G} = \begin{pmatrix} 0 & 0 & 0 & 0 \\ \theta_1 & \theta_3 & 0 & 0 \\ 0 & \theta_2 & \theta_3 & 0 \\ 0 & 0 & \theta_2 \phi \frac{s_T + \gamma K \exp(-1)}{\mu} & 0 \end{pmatrix}$ *and* $\bar{\boldsymbol{0}} = \begin{pmatrix} 0, & 0 \end{pmatrix}'$ *is positively invariant with respect to the flows of the system of equations (24)-(29).*

Proof. Using the procedure used to prove Proposition 1, it can easily be shown that

$$n(t) \leq \bar{n}$$

where

$$\bar{n} = \begin{pmatrix} s_T + \gamma K \exp(-1) & \frac{s_T + \gamma K \exp(-1)}{\mu} \end{pmatrix}'.$$

Substituting inequality (9) and $\bar{n}$ into equation of the virus at its different stages of the life cycle, yields

$$N(t+1) \leq \bar{G}N(t) + B\bar{n}. \tag{30}$$

We can prove by induction that $N(t) \leq \bar{G}^t N(0) + \sum_{i=0}^{t-1}(\bar{G}^i B\bar{n})$. It can then be shown that

$$\limsup_{t\to\infty} N(t) \leq (I-\bar{G})^{-1}B\bar{n}.$$

□

Equilibrium points

The equilibrium points (fixed points) are obtained by equating the right hand side to the left hand side of the system of equations (24)-(29). If we denote the equilibrium point by $\begin{pmatrix} D, & P, & Q, & V, & T, & T^* \end{pmatrix}'$, we have the following system of equations

$$\begin{aligned} D &= \beta_2 T^*, & (31)\\ P &= \theta_1 D + \theta_3 P, & (32)\\ Q &= \theta_2 P + \theta_3 Q, & (33)\\ V &= \theta_2 \phi Q T^*, & (34)\\ T &= s_T + T\gamma \exp\left(-\beta_2 T^* - \frac{T+T^*}{K}\right), & (35)\\ T^* &= T(1-\exp(-\beta_2 T^*)) + (1-\mu)T^*. & (36) \end{aligned}$$

Substituting eq (31) into eq (32) we have

$$P = \frac{\theta_1}{1-\theta_3}(\beta_2 T^*). \tag{37}$$

Eq (37) into eq (33) result in

$$Q = \frac{\theta_1\theta_2}{(1-\theta_3)^2}(\beta_2 T^*). \tag{38}$$

Substituting eq (38) into eq (34) we have

$$V = \frac{\theta_1\theta_2^2\phi}{(1-\theta_3)^2}(\beta_2 T^*)T^*. \tag{39}$$

$V = 0$ in (39) implies $T^* = Q = P = 0$. Substituting $T^* = 0$ into equations (35) and (36) and solving the two equation for T, we get T which is a solution of equation

$$T - s_T - T\gamma \exp\left(-\frac{T}{K}\right) = 0. \tag{40}$$

Proposition 6. *The disease free equilibrium point exist and is locally asymptotically stable for*

$$\left|\gamma\left(1-\frac{T}{K}\right)\exp\left(-\frac{T}{K}\right)\right|<1$$

and

$$|-\beta_2 T+1-\mu_T|<1$$

where T is a solution of eq (40).

Proof. The disease free equilibrium is given by

$$E_1=\begin{pmatrix} 0, & 0, & 0, & 0, & T, & 0 \end{pmatrix}',$$

where T is a solution of eq (40).

The Jacobian at E_1 is given by

$$J_{E_1}=\begin{pmatrix} 0 & 0 & 0 & 0 & 0 & \beta_2 \\ \theta_1 & \theta_3 & 0 & 0 & 0 & 0 \\ 0 & \theta_2 & \theta_3 & 0 & 0 & 0 \\ 0 & 0 & 0 & 0 & 0 & 0 \\ 0 & 0 & 0 & 0 & G1 & -\gamma\beta_2 T\exp(-\frac{T}{K}) \\ 0 & 0 & 0 & 0 & 0 & -\beta_2 T+1-\mu_T \end{pmatrix}.$$

All the eigenvalues of J_{E_1}, (0 (twice), G1, θ_3 (twice) and $-\beta_2\mathrm{T}^*+1-\mu$) have magnitudes less than one if and only if $|G1|<1$ and $|-\beta_2 T+1-\mu_T|<1$. □

The disease equilibrium point is obtained by equating the right hand side to the left hand side of the system of equations (24)-(29). If we denote the equilibrium point by $\big(D^*, \; P^*, \; Q^*, \; V^*, \; T^*, \; T$
we have the following system of equations

$$D^* = \beta_2 T^{**}, \tag{41}$$

$$P^* = \theta_1 D^*+\theta_3 P^*, \tag{42}$$

$$Q^* = \theta_2 P^*+\theta_3 Q^*, \tag{43}$$

$$V^* = \theta_2\phi Q^* T^{**}, \tag{44}$$

$$T^* = s_T+T^*\gamma\exp\left(-\beta_2 T^{**}-\frac{T^*+T^{**}}{K}\right), \tag{45}$$

$$T^{**} = T^*(1-\exp(-\beta_2 T^{**}))+(1-\mu)T^{**}. \tag{46}$$

Substituting eq (41) into eq (42) we have

$$P^*=\frac{\theta_1}{1-\theta_3}(\beta_2 T^{**}). \tag{47}$$

Substituting eq (47) into eq (43) result in

$$Q^* = \frac{\theta_1\theta_2}{(1-\theta_3)^2}(\beta_2 T^{**}). \tag{48}$$

Substituting eq (48) into eq (44) we have

$$V^* = \frac{\theta_1\theta_2^2\phi}{(1-\theta_3)^2}(\beta_2 T^{**})T^{**}. \tag{49}$$

Making T^* subject of formula in eq (46) we get

$$T^* = \frac{\mu T^{**}}{1-\exp(-\beta_2 T^{**})}. \tag{50}$$

Substituting this expression of T^* into eq (45) and simplifying the resulting equation we get

$$\mu T^{**} - s_T(1-\exp(-\beta_2 T^{**})) - \gamma\mu T^{**}\exp\left(-\beta_2 T^{**} - \frac{\mu T^{**}}{K(1-\exp(-\beta_2 T^{**}))} - \frac{T^{**}}{K}\right) = 0. \tag{51}$$

Solving for T^{**} is complex and we resort to graphical solutions. We let

$$f(T^{**}) = \mu T^{**} - s_T(1-\exp(-\beta_2 T^{**})) - \gamma\mu T^{**}\exp\left(-\beta_2 T^{**} - \frac{\mu T^{**}}{K(1-\exp(-\beta_2 T^{**}))} - \frac{T^{**}}{K}\right). \tag{52}$$

The cell-free and cell-associated viral spread model

The model that considers both forms transmission takes the form;

$$D_{t+1} = \beta_1 V_t + \beta_2 T_t^*, \tag{53}$$

$$P_{t+1} = \theta_1 D_t + \theta_3 P_t, \tag{54}$$

$$Q_{t+1} = \theta_2 P_t + \theta_3 Q_t, \tag{55}$$

$$V_{t+1} = \theta_2\phi Q_t T_t^*, \tag{56}$$

$$T_{t+1} = s_T + \gamma T_t \exp\left(-\beta_1 V_t - \beta_2 T_t^* - \frac{T_t + T_t^*}{K}\right), \tag{57}$$

$$T_{t+1}^* = T_t\left(1-\exp\left(-\beta_1 V_t - \beta_2 T_t^*\right)\right) + (1-\mu)T_t^*. \tag{58}$$

Proposition 7. *The system of equations (53)-(58) has a disease free equilibrium point given by*

$$E_3 = \begin{pmatrix} 0, & 0, & 0, & 0, & T^*, & 0 \end{pmatrix}',$$

where T^* is a solution of eq (20).

The Jacobian matrix at E_3 is given by

$$J_{E_3} = \begin{pmatrix} 0 & 0 & 0 & \beta_1 & 0 & \beta_2 \\ \theta_1 & \theta_3 & 0 & 0 & 0 & 0 \\ 0 & \theta_2 & \theta_3 & 0 & 0 & 0 \\ 0 & 0 & 0 & 0 & 0 & 0 \\ 0 & 0 & 0 & -\gamma\beta_1 T^* \exp\left(-\frac{T^*}{K}\right) & G1 & -\gamma\beta_2 T^* \exp\left(-\frac{T^*}{K}\right) \\ 0 & 0 & 0 & -\beta_1 T^* & 0 & -\beta_2 T^* + 1 - \mu \end{pmatrix}.$$

The eigenvalues are 0 (twice), θ_3 (twice), G1, and $-\beta_2 T^* + 1 - \mu$. All the eigenvalues have magnitudes less than one if and only if $|G1| < 1$ and $| - \beta_2 T^* + 1 - \mu| < 1$. Showing that the system of equations (53)-(58) has an endemic equilibrium is maybe difficult, we use permanence to show the existence of the disease equilibrium.

Definition 1. *The system (53)-(58) is permanent if there exist positive constants m and M which are independent of the solution of system (53)-(58), such that any positive solution $\{D_t, P_t, Q_t, V_t, T_t, T_t^*\}$ of system (53)-(58) satisfies*

$$m \leq \liminf_{t\to\infty}\{D_t, P_t, Q_t, V_t, T_t, T_t^*\} \leq \limsup_{t\to\infty}\{D_t, P_t, Q_t, V_t, T_t, T_t^*\} \leq M.$$

Lemma 1. *Every solution $\{D_t, P_t, Q_t, V_t, T_t, T_t^*\}$ of system (53)-(58) satisfies $\limsup_{t\to\infty} n(t) \leq \bar{n}$ and $\limsup_{t\to\infty} N(t) \leq (I - \bar{G})^{-1} B\bar{n}$, where $\bar{n}$, $\bar{G}$ are as previously defined.*

Proof. From the proof of Proposition 1 we get that

$$\limsup_{t\to\infty} T_t \leq s_T + \gamma K \exp(-1).$$

Using the same procedure as the one used in proving Proposition 1, we get

$$\limsup_{t\to\infty} T_t^* \leq \frac{s_T + \gamma K \exp(-1)}{\mu}$$

and

$$\limsup_{t\to\infty} N(t) \leq (I - \bar{G})^{-1} B\bar{n},$$

where $\bar{n}$, $\bar{G}$ are as previously defined. □

Lemma 2. *Every solution $\{D_t, P_t, Q_t, V_t, T_t, T_t^*\}$ of system (53)-(58) satisfies $h \leq \liminf_{t\to\infty} n(t)$ and $H \leq \liminf_{t\to\infty} N(t)$ where $h = (H_1\ H_2)$, $H = (1 - \tilde{G})Bh$,* $\tilde{G} = \begin{pmatrix} 0 & 0 & 0 & 0 \\ \theta_1 & \theta_3 & 0 & 0 \\ 0 & \theta_2 & \theta_3 & 0 \\ 0 & 0 & \theta_2\phi H_2 & 0 \end{pmatrix}$,

$H_1 = \gamma K \exp(-\beta_1 \bar{V} - \beta_2 \bar{T}^* - 1)$ *and*

$$H_2 = \gamma K \exp(-\beta_1 \bar{V} - \beta_2 \bar{T}^* - 1)\left(1 - \ln\left(\frac{\mu}{\beta_2 H_1}\right)\right) + \frac{\mu}{\beta_2} \ln\left(\frac{\mu}{\beta_2 H_1}\right).$$

Proof. According to Lemma 1, there exist a $t^* \in \mathbb{Z}^+$, such that

$$n(t) < \bar{n} + (\epsilon,\ \epsilon)',\ N(t) < \bar{N} + (\epsilon,\ \epsilon,\ \epsilon,\ \epsilon)',\ t \geq t^*$$

where $\bar{N} = (I - \bar{G})^{-1} B\bar{n}$. We first show that $\liminf_{t\to\infty} T_t \geq H_1$, where H_1 is to be determined. Assume that there exist a $t_0 \geq t^*$ such that

$$\begin{aligned} T_{t_0+1} &\geq s_T + \gamma T_{t_0} \exp\left(-\beta_1(\bar{V} + \epsilon) - \beta_2(\bar{T^*} + \epsilon) - \frac{T_{t_0}}{K}\right), \\ &\geq s_T + \gamma T_{t_0} \exp\left(-F - \frac{T_{t_0}}{K}\right), \\ &\geq \gamma T_{t_0} \exp\left(-F - \frac{T_{t_0}}{K}\right), \\ &\geq \gamma K \exp(-F - 1), \end{aligned}$$

where $-\beta_1(\bar{V} + \epsilon) - \beta_2(\bar{T^*} + \epsilon) = F$. We claim that

$$T_t \geq K \exp(-F - 1),\ \text{for t} \geq \text{t}_0.$$

By way of contradiction, assume there exist a $\tau_0 > t_0$ such that $T_{\tau_0} < \gamma K \exp(-F - 1)$. Let $\bar{\tau}_0 = t_0 + 2$ be the smallest number such that $T_{\bar{\tau}_0} < \gamma K \exp(-F - 1)$. The argument presented above produces a contradiction and this proves the claim. Thus

$$\liminf_{t\to\infty} T_t \geq \gamma K \exp(-F - 1).$$

Setting $\epsilon \to 0$ leads to

$$\liminf_{t\to\infty} T_t \geq H_1 = \gamma K \exp(-\beta_1 \bar{V} - \beta_2 \bar{T^*} - 1).$$

We now need to show that $\liminf_{t\to\infty} T^*_t \geq H_2$, where H_2 is to be determined. From the above steps we have that for $t^* \in \mathbb{Z}^+$,

$$n(t) < \bar{n} + (\epsilon,\ \epsilon)',\ N(t) < \bar{N} + (\epsilon,\ \epsilon,\ \epsilon,\ \epsilon)',$$

and $T_t \geq H_1 - \epsilon,\ t \geq t^*$. Assume that there exist $t_0 \geq t^*$ such that

$$\begin{aligned} T^*_{t_0+1} &\geq (H_1 - \epsilon)\left(1 - \exp\left(-\beta_1 V_{t_0} - \beta_2 T^*_{t_0}\right)\right) + (1-\mu)T^*_{t_0}, \\ &\geq (H_1 - \epsilon)\left(1 - \exp\left(-\beta_2 T^*_{t_0}\right)\right) + (1-\mu)T^*_{t_0}, && (59) \\ &\geq (H_1 - \epsilon)\left(1 - \exp\left(-\beta_2 T^*_{t_0}\right)\right) - \mu T^*_{t_0}, && (60) \\ &\geq (H_1 - \epsilon)\left(1 - \ln\left(\frac{\mu}{\beta_2(H_1 - \epsilon)}\right)\right) + \frac{\mu}{\beta_2} \ln\left(\frac{\mu}{\beta_2(H_1 - \epsilon)}\right). && (61) \end{aligned}$$

We claim that

$$T_t^* \geq H1_2, \text{ where } \mathrm{H1}_2 = (\mathrm{H}_1 - \epsilon)\left(1 - \ln\left(\frac{\mu}{\beta_2(\mathrm{H}_1 - \epsilon)}\right)\right) + \frac{\mu}{\beta_2}\ln\left(\frac{\mu}{\beta_2(\mathrm{H}_1 - \epsilon)}\right)$$

for $t \geq t_0$.

By way of contradiction assume there exists a $\tau_0 > t_0$ such that $T_{\tau_0}^* < H1_2$, then $\tau_0 \geq t_0 + 2$. Let $\bar{\tau}_0 = t_0 + 2$ be the smallest number such that $T_{\bar{\tau}_0}^* < H1_2$. The above argument produces $T_{\bar{\tau}_0}^* \leq H1_2$, a contradiction and this proves the claim. Thus $\liminf_{t\to\infty} T_t^* \geq H1_2$. Setting $\epsilon \to 0$ leads to

$$\liminf_{t\to\infty} T_t \geq H_2 = H_1\left(1 - \ln\left(\frac{\mu}{\beta_2 H_1}\right)\right) + \frac{\mu}{\beta_2}\ln\left(\frac{\mu}{\beta_2 H_1}\right)$$

We now show that $\liminf N(t) \geq H_3$ where H_3 is to be determined. We now have that $n(t) < \bar{n} + (\epsilon,\ \epsilon)'$, $N(t) < \bar{N} + (\epsilon,\ \epsilon,\ \epsilon,\ \epsilon)'$, and $T_t \geq H_1 - \epsilon,\ T_t^* \geq H_2 - \epsilon,\ t \geq t^*$.

Define $\hat{G} = \begin{pmatrix} 0 & 0 & 0 & 0 \\ \theta_1 & \theta_3 & 0 & 0 \\ 0 & \theta_2 & \theta_3 & 0 \\ 0 & 0 & \theta_2\phi(H_2 - \epsilon) & 0 \end{pmatrix}$, and $\hat{\mathrm{n}} = (\mathrm{H}_1 - \epsilon,\ \mathrm{H}_2 - \epsilon)'$. For any $t \geq 0$ we have that

$$N(t+1) = GN(t) + B\hat{n} \quad (62)$$

$$\geq \hat{G}N(t) + B\hat{n} \quad (63)$$

We can prove by induction that $N(t) \geq \hat{G}^t N(0) + \sum_{i=0}^{t-1} \hat{G}^i B\hat{n}$, from which it can be deduced that $\liminf_{t\to\infty} N(t) \geq (I - \hat{G})B\hat{n}$. Setting $\epsilon \to 0$ leads to $\liminf_{t\to\infty} N(t) \geq H_3 = (I - \tilde{G})B\hat{n}$. □

Proposition 8. *The system of equations (53)-(58) is permanent.*

Proof. The result follows from Lemma 1 and Lemma 2. □

The implication of this result is that an endemic equilibrium point exist.

References

[1] Guckenheimer J. and Holmes P. 1983. Nonlinear osscillations, Dynamical systems, and birfurcations of vector fields. Springer-Verlag.
